# High Antibodies to VAR2CSA in Response to Malaria Infection Are Associated With Improved Birthweight in a Longitudinal Study of Pregnant Women

**DOI:** 10.3389/fimmu.2021.644563

**Published:** 2021-06-16

**Authors:** Alistair R. D. McLean, D. Herbert Opi, Danielle I. Stanisic, Julia C. Cutts, Gaoqian Feng, Alice Ura, Ivo Mueller, Stephen J. Rogerson, James G. Beeson, Freya J. I. Fowkes

**Affiliations:** ^1^ Burnet Institute, Melbourne, VIC, Australia; ^2^ Centre for Tropical Medicine and Global Health, Nuffield Department of Medicine, University of Oxford, Oxford, United Kingdom; ^3^ Department of Immunology and Pathology, Monash University, Melbourne, VIC, Australia; ^4^ Department of Medicine at the Doherty Institute, University of Melbourne, Melbourne, VIC, Australia; ^5^ Papua New Guinea Institute of Medical Research, Madang, Papua New Guinea; ^6^ Institute for Glycomics, Griffith University, Southport, QLD, Australia; ^7^ Population, Health and Immunity Division, Walter and Eliza Hall Institute of Medical Research, Parkville, VIC, Australia; ^8^ Département Parasites et Insectes Vecteurs, Institute Pasteur, Paris, France; ^9^ Department of Microbiology, Monash University, Clayton, VIC, Australia; ^10^ Department of Infectious Diseases, Central Clinical School, Monash University, Melbourne, VIC, Australia; ^11^ Centre for Epidemiology and Biostatistics, University of Melbourne, Melbourne, VIC, Australia; ^12^ Department of Epidemiology and Preventive Medicine, Monash University, Melbourne, VIC, Australia

**Keywords:** VAR2CSA antibodies, birthweight, placental infection, Papua New Guinea, malaria in pregnancy (MiP), *Plasmodium falciparum*

## Abstract

**Introduction:**

Pregnant women have an increased risk of *P. falciparum* infection, which is associated with low birth weight and preterm delivery. VAR2CSA, a variant surface antigen expressed on the parasitized erythrocyte surface, enables sequestration in the placenta. Few studies have prospectively examined relationships between antibody responses during pregnancy and subsequent adverse birth outcomes, and there are limited data outside Africa.

**Methods:**

Levels of IgG against VAR2CSA domains (DBL3; DBL5) and a VAR2CSA-expressing placental-binding *P. falciparum* isolate (PfCS2-IE) were measured in 301 women enrolled at their first visit to antenatal care which occurred mid-pregnancy (median = 26 weeks, lower and upper quartiles = 22, 28). Associations between antibody levels at enrolment and placental infection, birthweight and estimated gestational age at delivery were assessed by linear and logistic regression with adjustment for confounders. For all outcomes, effect modification by gravidity and peripheral blood *P. falciparum* infection at enrolment was assessed.

**Results:**

Among women who had acquired *P. falciparum* infection at enrolment, those with higher levels of VAR2CSA antibodies (75^th^ percentile) had infants with higher mean birthweight (estimates varied from +35g to +149g depending on antibody response) and reduced adjusted odds of placental infection (aOR estimates varied from 0.17 to 0.80), relative to women with lower levels (25^th^ percentile) of VAR2CSA antibodies. However, among women who had not acquired an infection at enrolment, higher VAR2CSA antibodies were associated with increased odds of placental infection (aOR estimates varied from 1.10 to 2.24).

**Conclusions:**

When infected by mid-pregnancy, a better immune response to VAR2CSA-expressing parasites may contribute to protecting against adverse pregnancy outcomes.

## Introduction

*Plasmodium falciparum* infections during pregnancy are associated with low birth weight and preterm delivery (1). Pregnant women are at an increased risk of detected *P. falciparum* infection relative to non-pregnant women and are at greatest risk during their first pregnancy (2). The variant surface antigen VAR2CSA, expressed on the infected erythrocyte (IE) surface (3–5), mediates adhesion to chondroitin sulfate A (CSA) (6) and thus enables sequestration of IEs in the placenta (7). VAR2CSA is a specific variant of *P. falciparum* erythrocyte membrane protein 1 (PfEMP1). Primigravid women and men in malaria endemic settings generally have low levels of antibodies specific for VAR2CSA but levels increase among women with increasing gravidity (8–12). It has been suggested that the reduced risk of malaria infection in multigravid women relative to primigravid women can be partly explained by the acquisition of naturally acquired antibodies to VAR2CSA with each pregnancy, providing protection against placental *P. falciparum* infection and its adverse consequences (11). On this basis, efforts are underway to design a vaccine to induce immunity against VAR2CSA to protect against placental infection (13, 14). However, there is very limited evidence from population studies to indicate that VAR2CSA antibodies are protective against adverse maternal and birth outcomes (15), especially in Asia-Pacific where a large population is at risk of malaria.

Most cross-sectional studies investigating antibodies in women at delivery have not found significant protective associations between levels of VAR2CSA antibodies and birthweight (15–18) or gestational age at delivery (15, 16) and a limited number of studies have noted protective associations in subsets of women according to clinical outcome or gravidity (3, 19–22). As VAR2CSA antibodies arise in response to placental infection but are also putatively protective against placental infection and its adverse outcomes, cross-sectional studies are limited in their ability to determine the protective effect of VAR2CSA immune responses.

The majority of longitudinal studies have been undertaken in Africa and have not observed significant associations between VAR2CSA antibodies measured mid-pregnancy and birthweight (23–26) or gestational age at delivery (26, 27). However, the presence of malaria infection can confound these associations as they are associated with both VAR2CSA antibodies and birth outcomes. Indeed, one African study demonstrated a positive association between VAR2CSA antibodies and birthweight, but only in women who had peripheral blood *P. falciparum* infection at enrolment; no association was observed in *P. falciparum* negative women (27). The probability of malaria infection is heterogeneous across individuals and in populations of varying malaria transmission. Investigating the potential modification of *P. falciparum* infection on associations between VAR2CSA and birth outcomes is needed. In addition, investigations in other regions that experience a high burden of malaria in pregnancy but may differ from Africa with respect to parasite and host genetics and human behaviour, such as the Asia-Pacific (28), are required.

In this study, we investigated whether antibodies measured at the first visit to ANC were associated with protection against adverse pregnancy outcomes in a longitudinal study in a malaria-endemic region of Papua New Guinea. We evaluated antibodies to VAR2CSA expressed on the surface of IEs and to specific domains expressed as recombinant proteins. We hypothesized that women who have higher magnitude antibody responses when infected during pregnancy will have better pregnancy outcomes than those with lower responses. Because of the heterogeneity of exposure to malaria in populations, we investigated how infection status at enrolment in mid-pregnancy influenced protective associations.

## Materials and Methods

### Study Population and Sample Collection

The study was carried out in the malaria endemic province of Madang in Papua New Guinea (PNG) as described in detail previously (29). Between September 2005 and October 2007, 470 pregnant women >16 years of age attending their first antenatal care visit at Alexishafen Health Centre were recruited following written informed voluntary consent. Women were followed up at 30-34 weeks gestation, at delivery and 6-8 weeks postpartum. Gestational age was estimated from fundal height measurements. At enrolment, women received chloroquine (9 or 12 tablets, 150 mg base) and (when available) sulphadoxine pyrimethamine (500/25 mg, three tablets), followed by weekly chloroquine prophylaxis (two 150 mg tablets weekly), and ferrous sulphate 270 mg and folic acid 0.3 mg daily, according to local policy. Prophylaxis was not monitored. Inclusion criteria included no history of multiple births (e.g. past delivery of twins) and delivery complications, intention to deliver at the Alexishafen Health Centre, haemoglobin (Hb) >5g/dl and evidence of foetal movement. This study included 301 women who had delivery data and peripheral blood samples available at the enrolment visit for antibody analysis. Peripheral blood samples were collected at each visit and plasma and serum samples were separated and frozen. Placental blood and placental biopsy samples were collected at delivery if the delivery occurred at the clinic (n = 233). At each visit peripheral parasitemia was determined by microscopy on thick and thin blood films and *Plasmodium spp* confirmed by PCR. The presence of placental infection was determined by placental histology of fixed Giemsa-stained placental biopsies by light microscopy and samples were classified as no infection (no malaria parasites present) or active infection (presence of malaria parasites) as previously described (30, 31). Data on human genetic polymorphisms of South-East Asian Ovalocytosis (SAO), Complement Receptor 1 (CR1) and α+thalassaemia were available as described (29). Samples from malaria-naive residents of Melbourne, Australia were used as malaria naive controls in all assays. Male Madang samples were collected between 2001 and 2002 from Modilon Hospital, the Madang town clinic, and the Yagaum immunization service (32).

This study received ethical approval from the PNG Medical Research Advisory Council, the Melbourne Health Human Research Ethics Committee and Alfred Health Human Research Ethics Committee.

### Parasite Culture and Selection

The CS2 *P. falciparum* parasite line that binds to CSA and predominantly express the *var2csa* transcript (33–35) was used in this study. *P. falciparum* CS2 parasites were cultured in O+ human red blood cells (RBCs) in RPMI-HEPES culture medium supplemented with 5% pooled non-exposed human serum and 0.1% Albumax (32). Parasites were routinely selected for knob expression by gelatin floatation (36).

### Measurement of Antibody Levels to Recombinant Proteins

Antibody levels were determined for 3 VAR2CSA recombinant proteins representing 2 allelic variants; DBL5 (3D7), DBL5 (7G8) and DBL3 (7G8) by standard ELISA assays (37). All recombinant proteins were cloned and produced in *Pichia pastoris* (38). DBL5 and DBL3 recombinant proteins were selected because compared to other VAR2CSA domains they are highly immunogenic in natural infections and elicit some degree of cross-reactive and adhesion-blocking antibodies (25, 39–43), and also promote opsonic phagocytosis by monocytes (44). Antibodies to apical membrane antigen 1 were assessed as a broad measure of blood-stage immunity (45). Recombinant proteins were coated onto plates overnight at concentrations of 0.5 µg/ml followed by 2 hour incubation with sera samples (1/500) and 1 hour incubation with goat anti-human IgG conjugated to horseradish peroxidase (HRP) (Millipore) (1/2500). Reactivity was determined by measuring the optical density (OD) at 405 nm following the addition of ABTS [2,2’-azino-bis(3-ethylbenzothiazoline-6-sulphonic acid)] (Thermo Fisher Scientific) for fifteen minutes and the reaction stopped with the addition of 1% sodium dodecyl sulfate in PBS. Results are presented as ODs standardized to five positive control samples (individuals from the study identified as having high IgG reactivity to VAR2CSA during assay optimisation) run on each assay plate to account for inter-plate variability.

### Measurement of Antibody Levels to VAR2CSA on the Surface of IEs

We used the CS2 parasite line to measure antibody levels to VAR2CSA expressed on the surface of IEs. The CS2 parasite line predominantly expresses the *var2csa* transcript (32, 33), binds to CSA and is recognized by serum from malaria exposed pregnant women (46, 47). Total IgG reactivity to the surface of CS2 IEs was assessed by flow cytometry as previously described (36, 48). Total IgG binding for each sample was determined by subtracting the geometric Mean Fluorescence Intensity (MFI) of uninfected erythrocytes from that of IEs. Geometric MFI were then expressed as a percentage of the mean geometric MFI (Arbitrary Units) of pooled serum from five positive high responders.

### Statistical Analysis

Statistical analyses were performed using Stata Version 16.1 (StataCorp, College Station, TX, USA).

Descriptive statistics were used to describe the included women, categorical variables were summarised with proportions and frequencies; continuous variables were summarised with quartiles. Linear regression was used to assess the associations between the exposures (antibody levels) and outcomes [birth weight (grams) and estimated gestational age at delivery (weeks)]. Logistic regression was used to assess associations between antibody levels and odds of placental infection (defined as presence of *P. falciparum* parasites in placental histology). Potential confounders for all analyses were selected *a priori* using causal diagrams (49). All analyses were adjusted for the following confounders: *P. falciparum* infection at enrolment (from peripheral blood sample, diagnosis by light microscopy of blood smears with confirmation by PCR), gravidity (primigravid/multigravid), haemoglobin at enrolment (g/dl; determined using Hemocue), middle upper arm circumference (cm), SAO (yes/no; by PCR), alpha thalassaemia (yes/no; by PCR). In the birth weight regression model, sex of baby (male/female) was included as a covariate known to be independently predictive of the outcome.

For all models, the assumption of a linear association between antibody levels and outcome was assessed both visually and by testing regression models with categorical (groups cut at quartiles) and pseudo-continuous antibody variables by likelihood ratio tests. As there was no evidence of non-linearity of associations between antibodies and outcomes in all models, antibody measures were fitted as continuous exposures. To aid interpretation, coefficients were presented representing the difference in outcome of a high responder (75^th^ percentile) to a low responder (25^th^ percentile). To assess whether the relationship between antibody levels and the outcome of interest was modified by gravidity (primigravid/multigravid) or *P. falciparum* infection at enrolment detected by light microscopy (yes/no), likelihood ratio tests were performed comparing the model with and without interaction terms. Where evidence supporting an interaction was present (p < 0.1 for at least two pregnancy-specific antibody exposures) this model was presented in the main text; results from all models are reported in **Supplementary Material**.

## Results

The median age of women was 24 years (lower, upper quartiles = 21, 28); 115 (38%) women were primigravid (**Table 1**). Median estimated gestational age at enrolment was 26 (22, 28) weeks. Primigravid women were more likely to present with peripheral blood *P. falciparum* infection [49/115 (43%); by light microscopy) than multigravid women [54/186 (29%)]. There were 103 (34%) women who had *P. falciparum* infection. Women infected at enrolment were more likely to experience adverse birth outcomes than women uninfected at enrolment with a higher percentage of low birth weight births (20% *vs* 15%) and preterm deliveries (30% *vs* 18%) and were more likely to experience placental *P. falciparum* infection (65% *vs* 53%).

**Table 1:** Study population characteristics in women with and without microscopic peripheral *P. falciparum* infection at enrolment.

	Total (N=301)	Uninfected at enrolment (N=198)	Infected at enrolment (N=103)
**Enrolment demographics**			
Age (years)	24 (21, 28)	25 (21, 30)	23 (20, 27)
Gravidity			
Primigravid	115/301 (38%)	66/198 (33%)	49/103 (48%)
Multigravid	186/301 (62%)	132/198 (67%)	54/103 (52%)
Gestational age (weeks)	26 (22, 28)	26 (22, 28)	26 (22, 29)
Middle Upper Arm Circumference (cm)	22 (21, 24)	23 (21, 24)	22 (22, 23)
Smokes	60/300 (20%)	40/198 (20%)	20/102 (20%)
**Maternal genetics**			
South East Asian Ovalocytosis	41/301 (14%)	23/198 (12%)	18/103 (17%)
α+thalassaemia			
Normal	59/301 (20%)	40/198 (20%)	19/103 (18%)
Heterozygous	112/301 (37%)	72/198 (36%)	40/103 (39%)
Homozygous	130/301 (43%)	86/198 (43%)	44/103 (43%)
Complement Receptor 1 (exon 22)			
AA	30/301 (10%)	21/198 (11%)	9/103 (8.7%)
AG	120/301 (40%)	84/198 (42%)	36/103 (35%)
GG	151/301 (50%)	93/198 (47%)	58/103 (56%)
**Haemoglobin at enrolment**			
Haemoglobin (g/dl)	8.4 (7.6, 9.4)	8.7 (7.9, 9.7)	8.1 (7.2, 9.0)
Severe anaemia (<8g/dl)	111/301 (37%)	60/198 (30%)	51/103 (50%)
**Infections at enrolment**			
Peripheral *P. falciparum* ^a^	103/301 (34%)	0/198 (0%)	103/103 (100%)
**Infections at delivery**			
Peripheral *P. falciparum* ^a^	33/288 (11%)	22/190 (12%)	11/98 (11%)
Placental *P. falciparum* ^b^	132/233 (57%)	81/154 (53%)	51/79 (65%)
**Haemoglobin at delivery**			
Haemoglobin (g/dl)	9.4 (8.2, 10.3)	9.4 (8.1, 10.4)	9.2 (8.2, 10.2)
Severe anaemia (<8g/dl)	68/285 (24%)	45/188 (24%)	23/97 (24%)
**Birth Outcomes**			
Birth weight (kg)	2.9 (2.6, 3.2)	3.0 (2.6, 3.2)	2.8 (2.5, 3.0)
Low birth weight (<2500g)	50/299 (17%)	30/197 (15%)	20/102 (20%)
Gestational age	38 (37, 40)	38 (37, 40)	38 (36, 40)
Preterm delivery	63/285 (22%)	35/191 (18%)	28/94 (30%)

Data are presented as median (25^th^ percentile, 75^th^ percentile) and n/total (%) for categorical variables. ^a^Parasite positive by light microscopy, confirmed by PCR. ^b^Parasite positive by placental histology. Smoker missing for 1 participant, Middle Upper Arm Circumference at enrolment missing for 12 participants, Age missing for 10 participants.

### Antibodies to VAR2CSA Were Associated With Gravidity and Exposure

**Figure 1:**
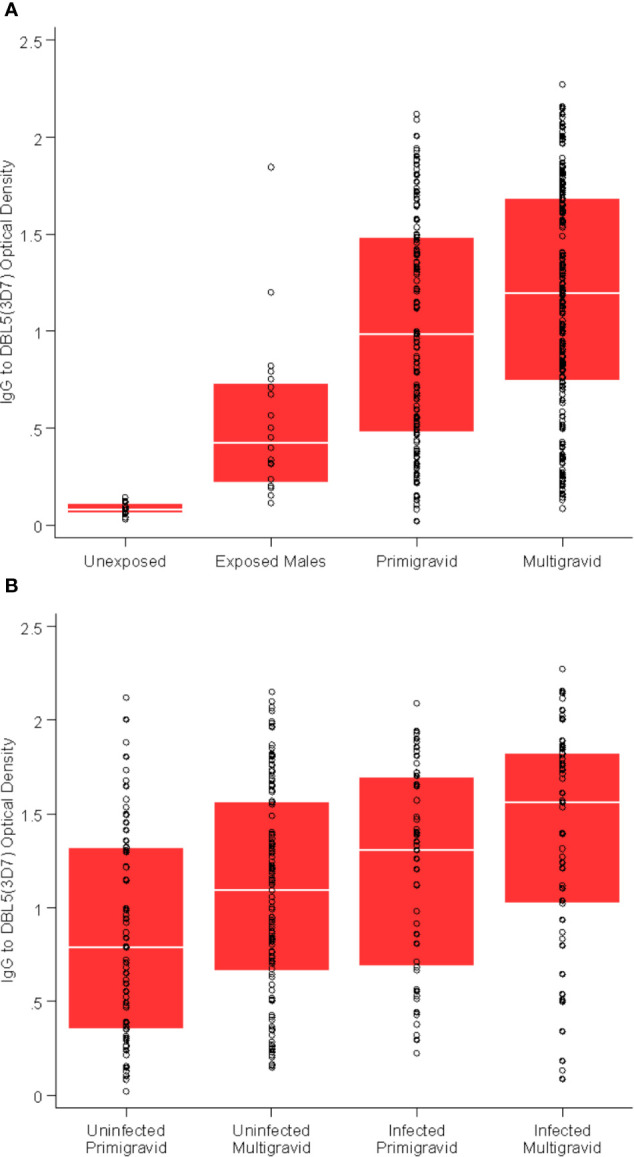
VAR2CSA-specific antibodies are associated with gravidity and exposure. **(A)** Total IgG to DBL5 (3D7) in unexposed Melbourne controls (n = 30); exposed males from Papua New Guinea (n = 20); primigravid women (n = 115); and multigravid (n = 186) women. p = 0.03 for multigravid versus primigravid; p < 0.01 for all other comparisons. **(B)** Total IgG to DBL5 (3D7) in primigravid uninfected at enrolment (n = 66); primigravid infected at enrolment (n = 49); multigravid uninfected at enrolment (n = 132); and multigravid infected at enrolment (n = 54). p = 0.02 and p = 0.08 for multigravid versus primigravid (uninfected and infected respectively); p < 0.01 for infected versus uninfected (primigravid and multigravid respectively).

Antibodies to VAR2CSA were assessed by quantifying IgG to recombinant DBL3 and DBL5 domains, and antibodies to the surface of CS2-IEs expressing VAR2CSA. VAR2CSA antibody levels were higher in multigravid women than primigravid women (p = 0.03), and higher in pregnant women compared to malaria-exposed men from the same population (p < 0.001) or malaria non-exposed adults (p < 0.001, **Figure 1A**). Women who were infected at enrolment had higher levels of VAR2CSA antibodies than women who were uninfected at enrolment (p < 0.001, **Figure 1B**).

### VAR2CSA IgG at Enrolment Was Differentially Associated With Placental Infection Among Women Infected Versus Non-Infected at Enrolment

**Figure 2:**
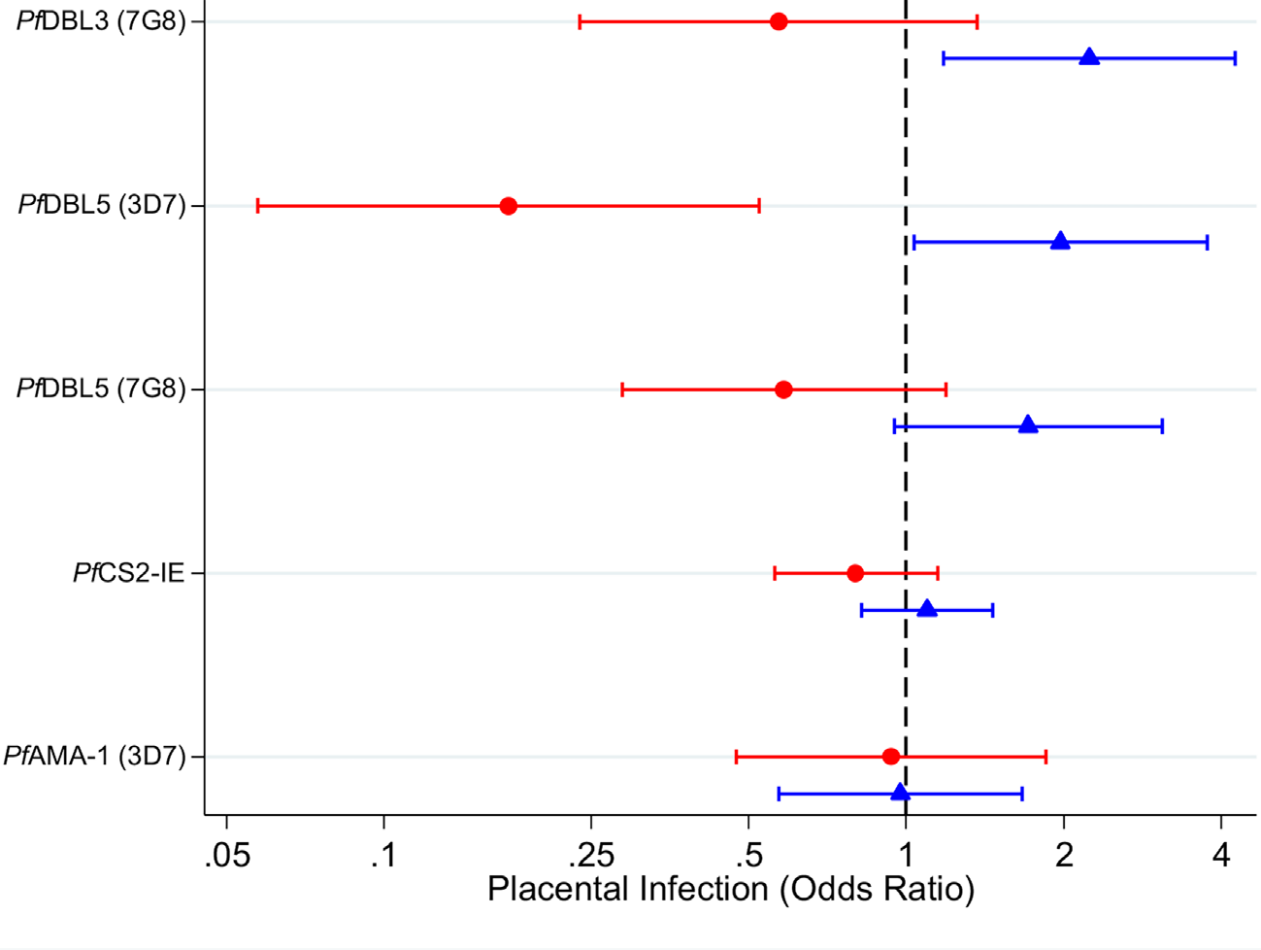
Adjusted odds ratios for placental *P. falciparum* infection in individuals with high antibody levels (75^th^ percentile) relative to individuals with low antibody levels (25^th^ percentile) in women infected at enrolment (red circles) and uninfected at enrolment (blue triangles). Capped bars indicate 95% confidence intervals. See **Supplementary Table 1** for a table with p values. See **Supplementary Tables 2** and **3** for estimates from models fitted with an interaction between antibodies and gravidity; and for estimates from models fitted without an interaction term.

There was evidence of effect modification of the association between VAR2CSA antibodies and odds of placental infection by *P. falciparum* infection at enrolment (**Supplementary Table S1**, likelihood ratio tests, p < 0.02 for DBL3 and DBL5; p = 0.11 for CS2-IE). Among women with infection detected at enrolment, women with higher levels of antibodies to VAR2CSA had lower adjusted odds of placental infection at delivery [**Figure 2**, Odds Ratio (OR) [95% confidence interval (CI)] = 0.6 (0.2,1.4); 0.2 (0.1,0.5); 0.6 (0.3,1.2); 0.8 (0.6,1.2) for DBL3 (7G8), DBL5 (3D7) and DBL5 (7G8) and CS2-IE respectively]. However, 95% CIs were wide and associations were generally not statistically significant [with the exception of DBL5 (3D7)]. In contrast, among women who were not infected at enrolment, higher levels of VAR2CSA antibodies were associated with an increased adjusted odds of placental infection (**Figure 2**, OR = 2.2 (1.2,4.3); 2.0 (1.0,3.8); 1.7 (1.0,3.1); 1.1 (0.8,1.5) for DBL3 (7G8), DBL5 (3D7) and DBL5 (7G8) and CS2-IE respectively). Levels of AMA1 antibodies (not specific to malaria in pregnancy), were not associated with odds of placental infection among infected women or uninfected women [OR = 0.9 (0.5,1.9) and 1.0 (0.6,1.7) respectively].

### VAR2CSA IgG Levels Associated With Increased Birth Weight in Women Infected During Pregnancy

Moderate evidence for effect modification by infection at enrolment was observed for some VAR2CSA antibodies.

**Figure 3:**
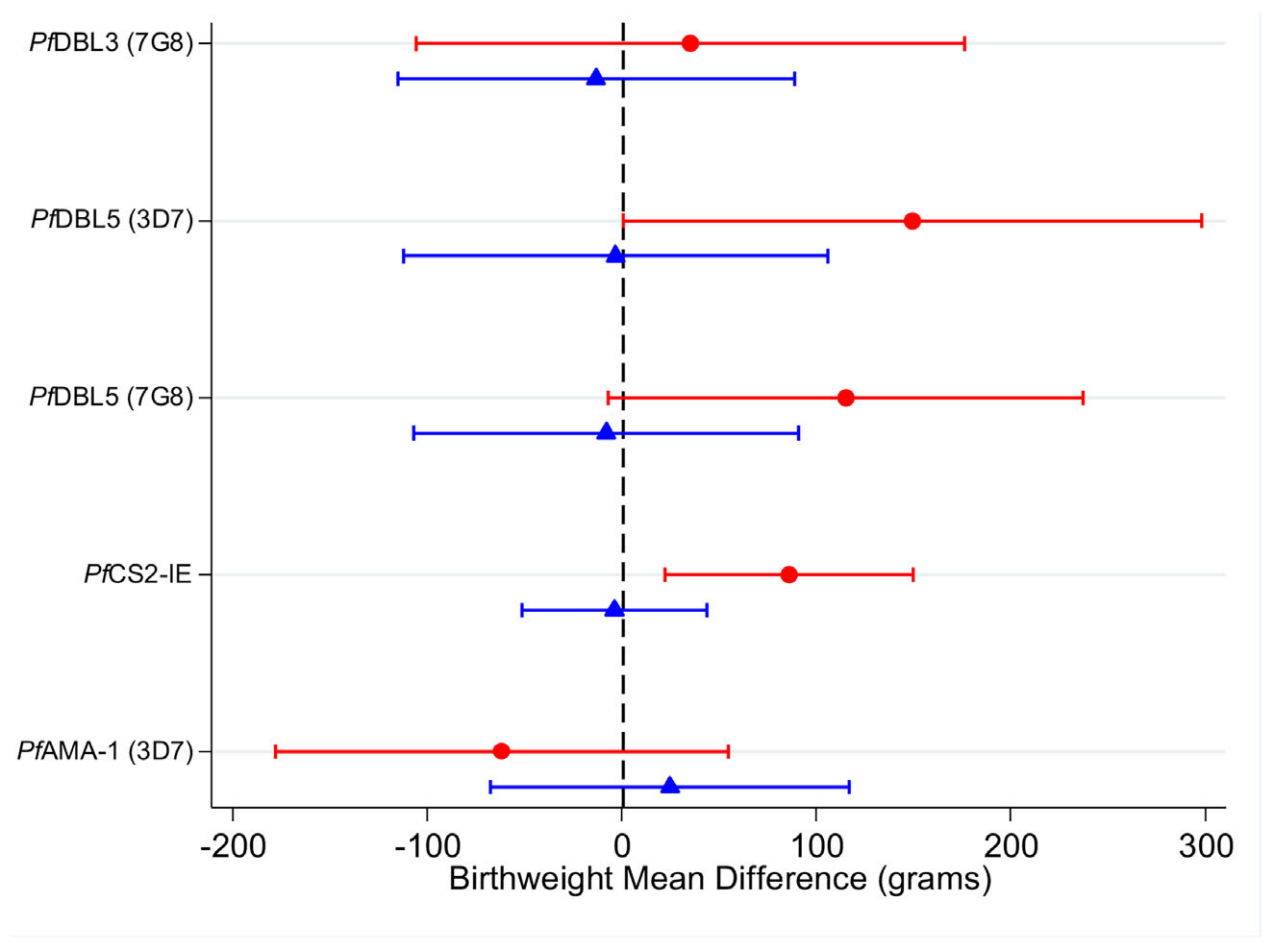
Adjusted mean difference in birthweight (grams) in individuals with high antibody levels (75^th^ percentile) relative to individuals with low antibody levels (25^th^ percentile) in women infected at enrolment (red circles) and uninfected at enrolment (blue triangles). Capped bars indicate 95% confidence intervals. See **Supplementary Table 4** for a table with p values. See **Supplementary Tables 5** and **6** for estimates from models fitted with an interaction between antibodies and gravidity; and for estimates from models fitted without an interaction term.

Among women infected at enrolment, higher levels of IgG to CS2-IEs, which represents IgG to full-length VAR2CSA (50), were associated with higher birth weight [+90g (20g-150g)] relative to women with lower antibody levels (**Figure 3** and **Supplementary Table S4**). Higher antibodies to VAR2CSA domains DBL5 (3D7 and 7G8) were also associated with higher birth weight [+150g (95% CI: 0g, +300g); +120g (-10g, +240g), respectively]. There was no clear association for DBL3 antibodies (+40g (-110g, +180g)). The estimated association between levels of antibodies to AMA1 was not significant (-60g (95% CI: -180g, +60g). Among women without infection detected at enrolment, the estimated associations between higher levels of VAR2CSA antibodies and birth weight were close to zero (estimates -10g to +0g). There was very little evidence to support effect modification by gravidity (**Supplementary Table S5**, likelihood ratio tests, p >0.3). Estimates of the associations between VAR2CSA antibodies and gestational age at delivery were of small magnitude and were not significant (**Supplementary Tables S7-9**).

## Discussion

In this study, we investigated the association between anti-VAR2CSA antibody responses and pregnancy outcomes, examining the influence of infection with *P. falciparum* at enrolment as well as gravidity on these associations. Women infected at enrolment and women who had been pregnant before had higher levels of anti-VAR2CSA antibodies at enrolment compared to women who were uninfected at enrolment, and women in their first pregnancy, respectively. Among women who were infected with *P. falciparum* at enrolment, those with higher VAR2CSA antibody levels had higher birthweight babies and lower odds of odds of LBW relative to those with low levels. These findings suggest that when women experience *P. falciparum* infection during pregnancy, the ability to generate higher levels of VAR2CSA antibodies may lead to better pregnancy outcomes.

Findings from previous studies investigating the associations between anti-VAR2CSA antibody responses and improved birth outcomes have been inconsistent and contradictory (3, 16–27, 51). Most of the literature reporting positive associations between anti-VAR2CSA antibodies and better birth outcomes have concentrated on particular subsets of infected women (3, 20–22, 27) or in particular strata of gravidity (19, 21), and often analyses were conducted as a cross-sectional study at delivery (15). Importantly, our analysis examined a cohort of pregnant women prospectively and formally tested for effect modification by *P. falciparum* infection and gravidity. This study supports the hypothesis that antibody responses to VAR2CSA are protective against lower birth weight, and possibly placental *P. falciparum* infection, but this protective association is only observed in women with evidence of *P. falciparum* infection at enrolment. Therefore, our findings suggest that women who generate higher antibody responses when infected in pregnancy have a reduced risk of low birthweight. That antibody levels in women with no peripheral blood *P. falciparum* infection at enrolment were instead associated with poor outcomes suggests that in this subset of women, antibodies are likely correlated with unmeasured recent infection, confounding any underlying protective association. Alternatively, it may be important to quantify antibodies during an active infection to better assess the nature and potential beneficial effects of antibody responses.

The strength of associations between antibody response to VAR2CSA and birth outcomes were broadly comparable across each measure of antibody response to VAR2CSA. It is beyond the scope of our immune-epidemiological study to be able to distinguish antibodies that have a truly protective role from antibodies that do not play a protective role but are associated with protection. However, a plausible role for antibodies to VAR2CSA in protective immunity is supported by the absence of any association between antibodies to AMA-1 and birth outcomes as well as observations that VAR2CSA antibodies can function to inhibit placental adhesion of PfIEs and promote phagocytosis (23, 52, 53). There may be other mechanisms that are not yet defined. A stronger association of antibodies specific for one domain relative to another domain may not indicate that these antibodies play a stronger direct role in protection. Strong associations with protection may indicate that these antibodies may be useful biomarkers for protective immunity. Further studies in multiple populations are needed to estimate the causal effect of single antibody responses and determine the importance of a repertoire of antibodies for maximal immunity. This knowledge will be valuable for informing vaccine approaches. Further assessment of functional mechanisms of protection (54, 55) for different epitopes or targets is warranted to clarify their role in the immune system in mediating protection. In this study, we only assessed antibodies to two VAR2CSA domains, DBL3 and DBL5. While these both are targets of acquired immunity and functionally-relevant antibodies (25, 39–44) and served the purpose of providing an assessment of responses to VAR2CSA in our study population, other domains have also been identified as targets of acquired immunity (25, 27). Detailed studies including an evaluation of all VAR2CSA domains and sub-domain could be valuable in future studies.

The longitudinal nature of this study allowed us to investigate a temporal relationship between the presence of antibodies mid-pregnancy at enrolment and subsequent adverse pregnancy outcomes, measured at delivery. Given that antibodies arise in response to exposure but may also serve a protective role against clinical malaria and adverse outcomes, it is difficult for cross-sectional studies (3, 16–22) to distinguish between women who have high antibody levels that reflect a recent infection and women who have high antibody levels that have successfully kept them infection free. In the absence of detailed exposure history, these studies will likely be subject to residual confounding by unmeasured exposure. A limitation of our study was measurement of *P. falciparum* exposure during, and prior to, the study; *P. falciparum* infection was only measured once using light microscopy and more frequent measurement of infection may have provided more accurate exposure data. Although these *P. falciparum* infections were observed during pregnancy, the acquisition of the infection will predate the moment it was detected, and some infections may have been present prior to conception. It is also possible that some women experienced undetected infections between recruitment and delivery and/or experienced and cleared infection prior to recruitment. This study was conducted in a setting where women received prophylaxis as part of their antenatal care which likely reduced *P. falciparum* infection and its negative effects during pregnancy. As such the magnitudes of protective effects of VAR2CSA immunity we observed may not be generalisable to other populations where malaria prophylaxis is not routinely given to pregnant women or in areas of different malaria endemicity.

In summary, we found that associations between VAR2CSA specific antibodies and birth outcomes varied across groups experiencing different levels of exposure. Among women who had already acquired infection by mid-pregnancy at enrolment, higher levels of antibodies to VAR2CSA were associated with a reduced risk of adverse outcomes. In contrast, among women uninfected at enrolment, anti-VAR2CSA antibodies may indicate women at increased risk of *P. falciparum* exposure and the adverse outcomes that follow. These differences in antibody associations due to exposure history might explain some of the conflicting reports in the published literature. Given the close relationship between exposure, antibodies and protection, ignoring heterogeneous exposure may obscure relevant protective associations. Future population studies should ensure the design and analysis accounts for the possibility of heterogeneous exposure.

## Data Availability Statement

The raw data supporting the conclusions of this article will be made available by the authors upon reasonable request and dependent on approval by the National Department of Health, Papua New Guinea.

## Ethics Statement

This study received ethical approval from the PNG Medical Research Advisory Council, the Melbourne Health Human Research Ethics Committee and Alfred Health Human Research Ethics Committee.Written informed consent to participate in this study was provided by the participants.

## Author Contributions

JB, FF, SR, and IM designed the research. AM, HO, DS, GF, and AU performed the research, AM and HO analysed the data, and AM, HO, JC, JB, and FF wrote the manuscript with input from all authors. All authors contributed to the article and approved the submitted version.

## Funding

This work was supported by the National Health and Medical Research Council (NHMRC: Research Fellowship 1166753 to FF, Project grant 575534, Program Grant 1092789 to JB. and SR, and Investigator Grant 1173046 to JB). Burnet Institute is supported by NHMRC Independent Research Institutes Infrastructure Support Scheme and the Victorian State Government Operational Infrastructure Support. JB, SR, IM, FF, HO, and JC are members of the NHMRC-funded Australian Centre for Research Excellence in Malaria Elimination.

## Conflict of Interest

The authors declare that the research was conducted in the absence of any commercial or financial relationships that could be construed as a potential conflict of interest.
