# Supplementary material for: High Antibodies to VAR2CSA in Response to Malaria Infection Are Associated With Improved Birthweight in a Longitudinal Study of Pregnant Women
Source: Front Immunol. 2021 Jun 16;12:644563. doi: 10.3389/fimmu.2021.644563 (PMC8242957; doi:10.3389/fimmu.2021.644563)
Supplement: Supplementary file 1 [file DataSheet_1.pdf]

Supplementary Materials

Supplementary Table S1. Adjusted odds ratios for placental *P. falciparum* infection in high antibody responders relative to low responders with effect modification by infection at enrolment status

	Infected				Uninfected				Likelihood ratio test (p value)
**Antibody**	**OR**	**LL**	**UL**	**p**	**OR**	**LL**	**UL**	**p**	
DBL3 (7G8)	0.57	0.24	1.37	0.208	2.24	1.18	4.25	0.014	0.009
DBL5 (3D7)	0.17	0.06	0.52	0.002	1.97	1.03	3.77	0.039	<0.001
DBL5 (7G8)	0.58	0.29	1.19	0.140	1.71	0.95	3.09	0.074	0.011
PfCS2-IE	0.80	0.56	1.15	0.227	1.10	0.82	1.46	0.531	0.107
AMA1 - (3D7)	0.94	0.47	1.85	0.848	0.97	0.57	1.67	0.925	0.911

Antibodies were modeled as a continuous exposure. A high antibody responder is considered to have the 75th percentile value for each antibody; a low antibody responder is considered to have the 25th percentile value for each antibody. LL = lower limit of 95% CI; UL = upper limit of 95% CI. OR – odds ratio.

Supplementary Table S2. Adjusted odds ratios for placental *P. falciparum* infection in high antibody responders relative to low responders with effect modification by gravidity (primigravid/multigravida)

	Primigravid				Multigravid				Likelihood ratio test (p value)
**Antibody**	**OR**	**LL**	**UL**	**p**	**OR**	**LL**	**UL**	**p**	
DBL3 (7G8)	0.86	0.37	1.99	0.719	1.84	0.98	3.46	0.059	0.147
DBL5 (3D7)	0.47	0.20	1.11	0.085	1.54	0.80	2.98	0.194	0.027
DBL5 (7G8)	0.88	0.40	1.91	0.740	1.34	0.78	2.31	0.291	0.367
PfCS2-IE	1.06	0.61	1.86	0.825	0.99	0.77	1.28	0.937	0.810
AMA1 - (3D7)	1.04	0.56	1.94	0.900	0.94	0.52	1.69	0.831	0.811

Antibodies were modeled as a continuous exposure. A high antibody responder is considered to have the 75th percentile value for each antibody; a low antibody responder is considered to have the 25th percentile value for each antibody. LL = lower limit of 95% CI; UL = upper limit of 95% CI. OR- odds ratio.

Supplementary Table S3. Adjusted odds ratios for placental *P. falciparum* infection in high antibody responders relative to low responders without effect modification

	All women			
**Antibody**	**OR**	**LL**	**UL**	**p**
DBL3 (7G8)	1.41	0.85	2.36	0.184
DBL5 (3D7)	1.00	0.59	1.69	0.995
DBL5 (7G8)	1.17	0.74	1.86	0.492
PfCS2-IE	1.00	0.79	1.27	0.987
AMA1 - (3D7)	0.99	0.64	1.52	0.946

Antibodies were modeled as a continuous exposure. A high antibody responder is considered to have the 75th percentile value for each antibody; a low antibody responder is considered to have the 25th percentile value for each antibody. LL = lower limit of 95% CI; UL = upper limit of 95% CI. OR – odds ratio

Supplementary Table S4. Adjusted mean difference in birth weight (grams) in high antibody responders relative to low responders with effect modification by infection at enrolment

	Infected				Uninfected				Likelihood ratio test (p value)
**Antibody**	**Birth weight (grams)**	**LL**	**UL**	**p**	**Birth weight (grams)**	**LL**	**UL**	**p**	
DBL3 (7G8)	+35	-106	+176	0.622	-13	-115	+89	0.800	0.572
DBL5 (3D7)	+149	+1	+298	0.049	-3	-112	+106	0.954	0.093
DBL5 (7G8)	+115	-7	+237	0.064	-8	-107	+91	0.877	0.110
PfCS2-IE	+86	+22	+150	0.008	-4	-51	+44	0.879	0.019
AMA1 - (3D7)	-62	-178	+55	0.299	25	-67	+117	0.596	0.236

Antibodies were modeled as a continuous exposure. A high antibody responder is considered to have the 75th percentile value for each antibody; a low antibody responder is considered to have the 25th percentile value for each antibody. LL = lower limit of 95% CI; UL = upper limit of 95% CI

Supplementary Table S5. Adjusted mean difference in birth weight (grams) in high antibody responders relative to low responders with effect modification by gravidity (primigravid/multigravida)

	Primigravid				Multigravid				Likelihood ratio test (p value)
**Antibody**	**Birth weight (grams)**	**LL**	**UL**	**p**	**Birth weight (grams)**	**LL**	**UL**	**p**	
DBL3 (7G8)	+8	-126	+142	0.904	+1	-102	+103	0.991	0.926
DBL5 (3D7)	+73	-70	+217	0.316	+36	-74	+146	0.517	0.672
DBL5 (7G8)	+73	-56	+202	0.267	+24	-70	+118	0.611	0.525
PfCS2-IE	+72	-27	+172	0.152	+20	-23	+62	0.361	0.319
AMA1 - (3D7)	-74	-178	+29	0.159	+55	-46	+157	0.286	0.072

Antibodies were modeled as a continuous exposure. A high antibody responder is considered to have the 75th percentile value for each antibody; a low antibody responder is considered to have the 25th percentile value for each antibody. LL = lower limit of 95% CI; UL = upper limit of 95% CI

Supplementary Table S6. Adjusted mean difference in birth weight (grams) in high antibody responders relative to low responders without effect modification

	All women			
**Antibody**	**Birth weight (grams)**	**LL**	**UL**	**p**
DBL3 (7G8)	+3	-80	+87	0.936
DBL5 (3D7)	+50	-40	+139	0.274
DBL5 (7G8)	+41	-38	+119	0.309
PfCS2-IE	+27	-12	+67	0.176
AMA1 - (3D7)	-8	-81	+65	0.825

Antibodies were modeled as a continuous exposure. A high antibody responder is considered to have the 75th percentile value for each antibody; a low antibody responder is considered to have the 25th percentile value for each antibody. LL = lower limit of 95% CI; UL = upper limit of 95% CI

Supplementary Table S7. Adjusted mean difference in gestational age at delivery (weeks) in high antibody responders relative to low responders with effect modification by infection at enrolment

	Infected				Uninfected				Likelihood ratio test (p value)
**Antibody**	**Birthweight (grams)**	**LL**	**UL**	**p**	**Birthweight (grams)**	**LL**	**UL**	**p**	
DBL3 (7G8)	-0.48	-1.23	+0.26	0.199	+0.11	-0.43	+0.65	0.682	0.187
DBL5 (3D7)	+0.28	-0.53	+1.10	0.494	+0.36	-0.23	+0.96	0.231	0.873
DBL5 (7G8)	-0.02	-0.68	+0.63	0.942	+0.25	-0.28	+0.78	0.349	0.500
PfCS2-IE	-0.07	-0.43	+0.30	0.723	+0.21	-0.05	+0.46	0.107	0.198
AMA1 - (3D7)	+0.02	-0.62	+0.65	0.960	+0.09	-0.41	+0.59	0.727	0.856

Antibodies were modeled as a continuous exposure. A high antibody responder is considered to have the 75th percentile value for each antibody; a low antibody responder is considered to have the 25th percentile value for each antibody. LL = lower limit of 95% CI; UL = upper limit of 95% CI

Supplementary Table S8. Adjusted mean difference in gestational age at delivery (weeks) in high antibody responders relative to low responders with effect modification by gravidity (primigravid/multigravida)

	Primigravid				Multigravid				Likelihood ratio test (p value)
**Antibody**	**Gestational age (weeks)**	**LL**	**UL**	**p**	**Gestational age (weeks)**	**LL**	**UL**	**p**	
DBL3 (7G8)	+0.01	-0.71	+0.73	0.986	-0.14	-0.68	+0.39	0.596	0.729
DBL5 (3D7)	+0.58	-0.20	+1.36	0.145	+0.20	-0.40	+0.80	0.516	0.423
DBL5 (7G8)	+0.23	-0.48	+0.94	0.524	+0.10	-0.39	+0.60	0.682	0.764
PfCS2-IE	+0.19	-0.49	+0.88	0.575	+0.12	-0.11	+0.34	0.313	0.826
AMA1 - (3D7)	-0.08	-0.65	+0.49	0.774	+0.19	-0.35	+0.74	0.487	0.481

Antibodies were modeled as a continuous exposure. A high antibody responder is considered to have the 75th percentile value for each antibody; a low antibody responder is considered to have the 25th percentile value for each antibody. LL = lower limit of 95% CI; UL = upper limit of 95% CI

Supplementary Table S9. Adjusted mean difference in gestational age at delivery (weeks) in high antibody responders relative to low responders without effect modification

	All women			
**Antibody**	**Gestational age (weeks)**	**LL**	**UL**	**p**
DBL3 (7G8)	-0.09	-0.53	+0.35	0.681
DBL5 (3D7)	+0.34	-0.15	+0.82	0.175
DBL5 (7G8)	+0.14	-0.27	+0.56	0.499
PfCS2-IE	+0.12	-0.09	+0.34	0.262
AMA1 - (3D7)	+0.06	-0.33	+0.45	0.761

Antibodies were modeled as a continuous exposure. A high antibody responder is considered to have the 75th percentile value for each antibody; a low antibody responder is considered to have the 25th percentile value for each antibody. LL = lower limit of 95% CI; UL = upper limit of 95% CI
